# Recent Progress on Flexible Electronic Devices Based on Buckled Structures with Polymeric Substrates

**DOI:** 10.3390/polym18151887

**Published:** 2026-07-31

**Authors:** Dawei Dong, Bin Hu, Simin Zhao, Kun Dai, Chaojun Gao, Guoqiang Zheng, Chuntai Liu, Changyu Shen

**Affiliations:** School of Materials Science and Engineering, National Engineering Research Center for Advanced Polymer Processing Technology, Zhengzhou University, Zhengzhou 450001, China; ddw@stu.zzu.edu.cn (D.D.); bhu_pfpg@163.com (B.H.); smzhao_pfpg@163.com (S.Z.); kundai@zzu.edu.cn (K.D.); ctliu@zzu.edu.cn (C.L.); shency@zzu.edu.cn (C.S.)

**Keywords:** buckled structures, flexible electronic devices, emerging scenarios

## Abstract

Recently, flexible electronics have attracted widespread attention in personalized health monitoring, soft robotics, and smart human-machine interactions due to intrinsic high stretchability. Among them, constructing buckled structures in flexible devices is one of the most effective strategies to achieve flexibility and stretchability. Flexible electronic devices based on buckled structure (FEDB) have gained significant research progress, owing to their outstanding advantages such as simple fabrication processes, excellent structural stability, and broad applicability. Furthermore, its application areas are expanding to emerging scenarios, including the human body, underwater environments, the oceans, and space. However, there are few systematic reviews concerning their progresses, although researchers show increasing interest in the emerging applications of FEDB. This review summarizes recent research progress in FEDB. First, this review explains the buckled instability mechanism, listing the common conductive and substrate materials. The polymeric substrates discussed mainly include PDMS, TPU, SBS, PC, and hydrogel, which provide the flexibility and deformability required for FEDB. In addition, this review summarizes several methods for constructing buckled structures, including prestretch-release, solvent swelling, thermal, mold, and 3D printing as well as techniques for controlling morphology. Second, this review summarizes the applications of FEDB, such as flexible electrodes, strain and pressure sensors, and energy devices. Particularly, it provides a detailed introduction to the expansion of emerging scenarios, involving underwater monitoring, in vitro and in vivo physiological signal detection, human-machine interactions, and portable capsule devices. Finally, this review points out the current challenges of FEDB, including long-term service stability, adaptability to extreme environments, conformal attachment to complex curved surfaces, and large-scale manufacturing.

## 1. Introduction

Rigid electronic devices play a significant role in modern industrial and technological development [1]. However, the inherent high Young’s modulus of rigid electronic devices has limited its application scenarios in the field of flexible materials (such as human tissue, complex 3D (3D) surfaces, and dynamic deformation environments) with the rapid rise of the Internet of Things (IoT) [2], personalized medicine [3], wearable devices [4], and soft robotics [5]. Developing flexible electronic devices that can be bent, compressed, or even stretched has attracted widespread attention and shows tremendous potential [6].

Currently, there are two main strategies for fabricating flexible electronic devices [7]. The first strategy is to develop intrinsically flexible conductive materials through molecular design or physical blending. Examples include liquid metals, ionic liquids, conductive polymers, and various types of conductive elastomer composites [8]. The as-fabricated electronic devices can achieve a balance between high performance and flexibility. However, they inevitably experience performance degradation during long-term service [9]. The second strategy is to design microstructural or macrostructural mechanical structures, enabling inherently rigid materials (such as metal films, graphene, transition metal carbonitrides, MXene, etc.) to gain flexibility or even stretchability [7]. The common structural designs include porous, mesh, buckled, and cut-paper structures [10,11,12,13,14]. Although the above structural design strategies have been proposed to achieve flexibility in electronic devices, different structures involve trade-offs in terms of fabrication and device performance. For example, cut-paper structures typically achieve high stretchability, but stress concentration tends to occur at the cut edges, and their fabrication often relies on relatively complex patterning processes. Porous structures can provide lower stiffness and good breathability, but their internal void network may compromise the stability of the device under severe mechanical deformation. Mesh structures can achieve good conformity on curved surfaces, but their complex fabrication processes limit scalable application.

**Figure 1:**
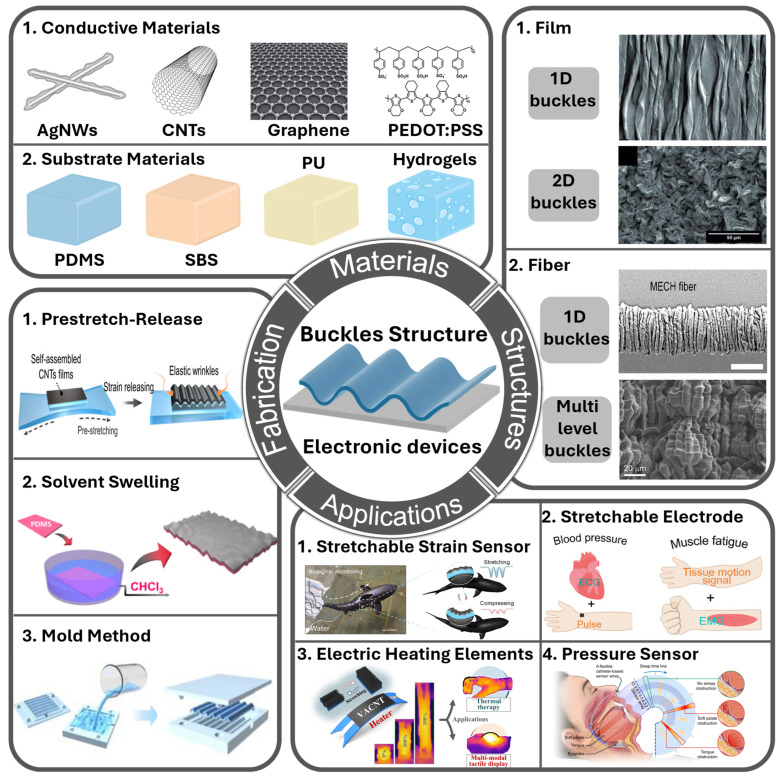
Schematic diagram of a FEDB. Illustrations reproduced from the following sources. Silver nanowires (AgNWs) and poly(3,4-ethylenedioxythiophene): poly(styrene sulfonate) (PEDOT: PSS): Reproduced with permission [28]. Copyright 2025 American Chemical Society. Prestretch-release: Reproduced with permission [27]. Copyright 2025 American Chemical Society. Solvent Swelling: Reproduced with permission [19]. Copyright 2016 American Chemical Society. Mold method: Reproduced with permission [21]. Copyright 2025 Wiley-VCH. Stretchable Strain Sensor: Reproduced with permission [27]. Copyright 2025, American Chemical Society. Stretchable Electrode: Reproduced with permission [29]. Copyright 2025, Wiley-VCH. Electric Heating Element: Reproduced with permission [30]. Copyright 2025, American Chemical Society. Pressure Sensor: Reproduced with permission [25]. Copyright 2024, Springer Nature. Film 1D buckles, Film 2D buckles: Reproduced with permission [20]. Copyright 2018 Wiley-VCH. Fiber 1D buckles: Reproduced with permission [31]. Copyright 2022 American Chemical Society. Fiber multi-level buckles: Reproduced with permission [32]. Copyright 2015 American Association for the Advancement of Science.

Among them, designing buckled structures is one of the most effective routes to achieve flexibility and stretchability of electronic devices, offering advantages such as simplicity of manufacture, structural stability, and wide applicability. The development of buckles originates from the modulus difference between different layers upon external forces [15]. When the device is subjected to compressive stress beyond the critical threshold, the rigid layer with a high Young’s modulus will undergo out-of-plane buckled instability, subsequently spontaneously forming a periodic wavy morphology [16]. Therefore, the electronic devices fabricated based on this structure can accommodate strain through the unbuckling of periodic buckling when subjected to external tension, bending, or twisting, thereby protecting the functional layer from damage. Various methods, including uniaxial stretching [17], biaxial stretching [18], solvent swelling [19], thermal [20], mold [21], and 3D printing [22], have been proposed to construct buckled structures with different morphologies and functions on polymer films or fibers. The wavelength, amplitude, orientation, and multi-level morphology of the buckles can be precisely controlled [23] and are widely applied in areas such as flexible electrodes, conductive interconnect components, and flexible sensors (Figure 1).

Flexible electronic devices based on buckled structure (FEDB) have achieved significant progress in flexibility and functionality. In recent years, the application scenarios of FEDB have gradually expanded to the fields of healthcare [24], health monitoring [25], human–machine interfaces [26], and underwater monitoring [27]. However, there remain no systematic reviews of these progresses, although researchers show growing interest in the emerging applications of FEDB. Additionally, FEDB still presents key challenges, including insufficient environmental stability, performance degradation over time, and relatively high manufacturing costs. Therefore, it is essential to summarize and review the latest studies on FEDB.

In view of this, this review outlines recent research progress on FEDB. Firstly, this review explains the instability mechanism of buckles, listing the common conductive and substrate materials and summarizing several methods for constructing buckles. Subsequently, this paper reviews the applications of FEDB, such as flexible electrodes, strain and pressure sensors, and energy devices. In particular, it provides a detailed introduction to the expansion of emerging scenarios, involving underwater monitoring, in vitro and in vivo physiological signal detection, human–machine interactions, and portable capsule devices. Finally, this review points out the current challenges of FEDB, including long-term service stability, adaptability to extreme environments, conformal attachment to complex curved surfaces, and large-scale manufacturing.

## 2. Fabrication of FEDB

As an effective way for enhancing the ductility of functional layers, designing buckled structures is widely used in the fabrication of flexible electronic devices. In particular, the selection of functional and substrate materials is critical to the buckled morphology and the performance of electronic devices. Additionally, by using specific methods to control the degree of prestressing and the adhesion between the functional layer and substrate, buckles with different morphologies and characteristic scales can be precisely fabricated. This section summarizes the mechanisms underlying buckling in bilayer systems, as well as the conductive and substrate materials commonly used in FEDB. In addition, it, in detail, summarizes the fabrication methods for buckled structures.

### 2.1. Formation Mechanism of Buckles

Taking a simple two-layer system (i.e., one with a thin non-flexible layer and a thick, soft, flexible support layer) as an example, the formation of a buckled structure is essentially due to the difference in modulus between the two layers, which results in different degrees of recovery and contraction. When the macroscopic compressive strain imposed on the system exceeds a certain critical value, the flat surface spontaneously becomes unstable and bends out of plane to minimize the system’s total potential energy, thereby evolving into regular sinusoidal buckles [16].

This instability process follows predictable mechanical patterns. The critical instability strain ($\epsilon_{c}$) and initial characteristic wavelength ($\lambda_{0}$) of the system depend on the mismatch in the mechanical properties of the material and the film thickness (h). The physical relationship can be described as (1) and (2) [15,33]:

(1) $$\epsilon_{c}=\frac{1}{4}{\left(\frac{3E_{s}}{E_{f}}\right)}^{2/3}$$

(2) $$\lambda_{0}=2\pi h{\left(\frac{E_{f}}{3E_{s}}\right)}^{1/3}$$

where $\bar{E_{f}}$ and $\bar{E_{s}}$, respectively, represent the plane strain moduli of the film and substrate. Since the modulus of the rigid layer in a bilayer system is often several orders of magnitude higher than that of the flexible substrate, $\epsilon_{c}$ is very small, leading to macroscopic buckling under minimal compressive stress. These explicit mechanical mapping relationships are widely used to fabricate buckles of various shapes and scales.

When electronic devices undergo stretching or bending, they can accommodate strain by expanding or contracting the buckled waveform, allowing the conductive layer to deform to a certain extent, unlike traditional rigid conductors, which are prone to breaking or losing their conductivity. Designing buckled structures provides inherently rigid electronic devices with sufficient flexibility to adapt to complex deformations and stresses [34]. Khang et al. [35] transferred ultra-thin single-crystal silicon nanoribbons onto a pre-stretched rubber substrate and successfully fabricated a classic buckled silicon structure by releasing the prestress. This study confirms that even intrinsically rigid semiconductor materials can utilize buckled structures to prevent mechanical fracture and electrical failure under high tensile strain, enabling inorganic materials to be used in the fabrication of flexible electronic devices. Furthermore, buckled morphology can be facilely tuned by multidimensional compression. Zang et al. [36] have successfully fabricated 2D buckled structures by transferring graphene onto a biaxially pre-stretched elastomeric substrate and releasing the prestress in a specific sequence. The fabricated devices can not only withstand bidirectional tensile strain but also exhibit hydrophobic properties. Kim et al. [33] found that, under biaxial compressive stress, buckles propagate along the surface and connect to other buckles, delineating individual closed domains. When compression is continuously applied, a repetitive buckle-to-fold transition occurs within these individual domains, further subdividing each domain over multiple generations. This sequential and orderly dynamic for domain division continues until it generates a complex, hierarchical network of buckles. Takei et al. [37] found that, subjected to high biaxial compression, polydimethylsiloxane (PDMS) films bulge outwards (upwards) from the substrate to form ‘ridges’, with relatively flat regions of film lying between them. In addition, Ohzono et al. [38] proposed a method for designing surface pre-patterns by utilizing self-assembled 2D microsphere arrays as lithographic masks. When the lateral periodicity of the lithographic pattern closely matches the characteristic wavelength of the micro-buckles, a novel directional order originating from the hexagonal packing of the microspheres is induced. This approach of introducing surface patterns with matching length scales prior to buckle formation effectively guides and restricts the evolutionary direction of the surface micro-buckles, thereby generating highly ordered, anisotropic buckled structures.

### 2.2. Materials for the Preparing FEDB

Based on the structure of FEDB, the materials can be divided into two categories: the substrate material and the conductive material [10]. Among them, conductive materials can perform various functions based on their inherent properties, such as heat generation, light emission, and transparency. The substrate material primarily provides flexibility that can further support the electronic devices [39]. In view of these, appropriate conductive materials and substrate materials should be selected based on the desired applications to ensure that the overall performance of FEDB is fully taken into account.

#### 2.2.1. Conductive Materials

The conductive materials commonly used in FEDB are primarily divided into metallic materials, carbon-based materials, and conductive polymers [8]. Table 1 lists their corresponding parameters, involving Young’s modulus, fracture strain, and electrical conductivity. Among these, metallic materials include conductive metals such as gold, silver, and copper, which are primarily used to manufacture electrodes. With continuous technological advancements, conductive nanoparticles and nanowires have attracted significant interest due to their excellent electrical conductivity, optoelectronic properties, and mechanical properties. In particular, the 1D linear structure and nanoscale dimension of metal nanowires (such as AgNWs and CuNWs) endow them with an exceptionally high aspect ratio. Therefore, it maintains good electrical conductivity even under bending and tensile deformation [40]. Wu et al. [41] deposited AgNWs on pre-stretched PDMS, releasing the pre-stress to create buckled structures on the PDMS surface. Using this method, flexible sensors and stretchable thermochromic devices with a wide detection range have been successfully fabricated.

Carbon-based materials, such as 1D carbon nanotubes and 2D graphene, exhibit characteristics including high carrier mobility, excellent electrical and thermal conductivity, high light transmittance, large specific surface area, high Young’s modulus, and outstanding mechanical flexibility. As a result, they are widely used in the field of FEDB fabrication. Yu et al. [58] deposited carbon nanotubes (CNTs) onto a pre-stretched PDMS substrate, releasing the pre-stress to successfully form buckled structures on its surface. The fabricated fibers maintain stable electrical resistance even under high tensile strain, which can be applied to stretchable electrodes, energy storage, and energy harvesting. Tang et al. [24] used a template-based casting method to fabricate a graphene layer with a buckled morphology on PDMS substrate, and the buckled morphology could be controlled. Pressure sensors prepared by this method can successfully monitor a wide range of signals, from weak stimuli to high pressure, such as detecting faint gas and foot pressure. In addition, it can be applied to tactile imaging and Braille recognition, holding significant potential for use in prosthetics and intelligent robotics.

Conductive polymers are a classification of electrically conductive polymeric materials characterized by a conjugated π-electron structure. The molecular chains of conductive polymers contain conjugated double-bond structures, in which single and double bonds alternate. This conjugated structure allows electrons to move relatively freely along the molecular chain, thereby enabling a certain degree of electrical conductivity. Common conductive polymers include polyaniline (PAn), polyacetylene (PAc), polypyrrole (PPy), and polythiophene (PT). Among these, PT serves as a highly representative conductive polymer. PEDOT is a polymer formed by the polymerization of 3,4-ethoxythiophene monomers, which features a delocalized conjugated π-electron system in its molecular chain. When an electric field is applied, these delocalized π electrons can move along the molecular chain, generating an electric current. PSS is a polyelectrolyte that can bind to PEDOT molecules through electrostatic interactions to form a stable complex, thereby enabling PEDOT to disperse evenly in water, which makes it easier to prepare films and devices. Kwon et al. [59] transferred a silver nanowire network onto a uniaxially prestrained substrate, applying solvent annealing during strain release to form a wavy nanowire structure. Subsequently, PEDOT: PSS and an ionic liquid were sequentially spin-coated onto this network, followed by thermal annealing, to fabricate a stretchable composite transparent electrode for organic solar cells.

It is worth noting that, in recent years, MXene has also been widely used for preparing flexible electronic devices. MXene is in a family of 2D transition metal carbides and/or nitrides with a 2D layered structure. This structure results in relatively low electrical resistance within the layer. In a 2D plane, transition metal atoms are linked to C or N atoms through chemical bonds, forming a continuous electron cloud. Electrons can move relatively freely in these continuous electron clouds, thereby enabling intralayer electron conduction. Similar to graphene, the 2D structure of MXene provides an efficient pathway for electrons, allowing them to move rapidly within the layers and resulting in excellent electrical conductivity. Wang et al. [60] deposited MXene/graphene onto pre-stretched PDMS, and a conductive layer with a buckled morphology was obtained after pre-strain was released. Such a bulked structure can be successfully assembled into a strain sensor with a wide detection range and excellent cycling stability. Such a sensor can accurately detect various human movements, such as blinking, speaking, bending fingers, and bending the wrist.

#### 2.2.2. Flexible Substrate Materials

Flexible polymer matrices can endure complex mechanical deformations, determining the mechanical and physical properties of devices, which are essential for the fabrication of FEDB [15,35]. Common flexible substrate materials include polyimide (PI), polyvinyl alcohol (PVA), and polyethylene terephthalate (PET), which provide a certain degree of flexibility and protection for FEDB. In addition, thermosetting elastomers such as PDMS are widely used due to providing higher tensile strength. When thermosetting elastomers cure, molecules from the precursor solution form crosslinking with the crosslinking agent molecules, creating a complex, interconnected 3D crosslinking network. By depositing an aluminum film onto PDMS with a buckled pattern, Wu et al. [61] achieved a controllable surface topography that meets the requirements for precise buckled structures in flexible electronic devices.

In addition, there are thermoplastic block copolymer elastomers, such as styrene-ethylene-butylene-styrene (SEBS), which are polymeric materials capable of plastic deformation at high temperatures. Its components include a hard segment and a soft segment. Common thermoplastic block copolymer elastomers include styrene-butadiene-styrene (SBS) and thermoplastic polyurethane (TPU). Liu et al. [32] deposited CNT sheets onto pre-stretched SBS substrate fibers, releasing the pre-stress to create a buckled structure on the fiber surface. Based on this, they fabricated flexible electrodes with high resistance insensitivity and a long service life. By using the swelling method, Zhang et al. [62] created a buckled structure on the surface of TPU fibers and deposited chitosan (CTS) and MXene, successfully fabricating a flexible strain sensor with high sensitivity and a wide measurement range.

It is worth noting that FEDB has been increasingly widely applied in the fields of tissue engineering, wearable technology, and implantable medical devices. FEDB-based flexible hydrogels have attracted significant attention because hydrogels’ mechanical and physicochemical properties closely resemble those of human soft tissues (such as skin and cardiac muscle) [63,64]. Hydrogels, derived from natural or synthetic materials, show a 3D crosslinking polymer network primarily through chemical and physical crosslinking [65]. Through electrochemical methods, Qiu et al. [64] fabricated microscopic buckle patterns on the surface of the hydrogel of PAM@SA, whose pressure sensor capable of detecting faint vibrations has been successfully fabricated.

### 2.3. Methods for Buckle Formation

**Figure 2:**
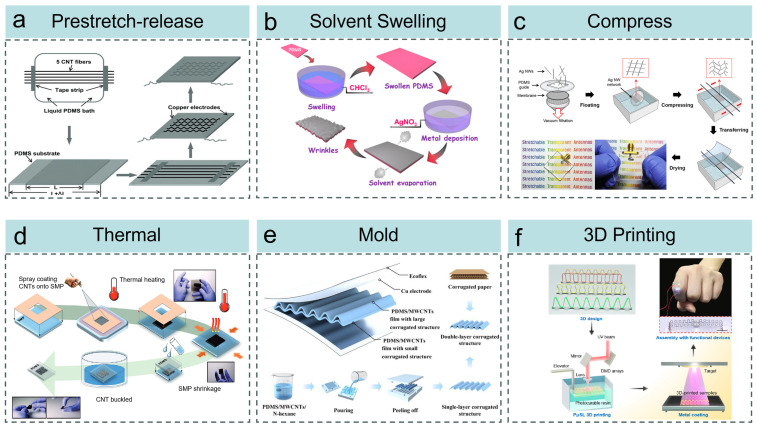
Common methods for fabricating buckles. (**a**) Carbon nanotube fibers were transferred onto a PDMS substrate with a pre-applied strain, and then releasing the strain resulted in a buckled structure. Reproduced with permission [17]. Copyright 2013 Wiley-VCH. (**b**) A buckled structure was obtained by swelling PDMS in chloroform and then depositing silver. Reproduced with permission [19]. Copyright 2016 American Chemical Society. (**c**) Compressing a network of AgNWs floating in water to form a buckled structure. Reproduced with permission [66]. Copyright 2016 American Chemical Society. (**d**) Heating a shape-memory polymer (SMP) substrate to induce bidirectional contraction of the carbon nanotube film, resulting in a buckled structure. Reproduced with permission [20]. Copyright 2018, Wiley-VCH. (**e**) After thoroughly mixing PDMS and multi-walled carbon nanotubes (MWCNTs), the mixture was poured into a moldboard to form a corrugated structure resembling corrugated cardboard. Reproduced with permission. [21] Copyright 2025, Wiley-VCH. (**f**) 3D printing of a CAD model using projection microstereolithography (PµSL) to produce a buckled structure. Reproduced with permission [22]. Copyright 2021 Wiley-VCH.

At present, researchers have developed various methods of fabricating buckled structures according to the properties of conductive and substrate materials, as well as the specific requirements of flexible electronic devices. As shown in Figure 2a–f, common methods for fabricating buckles include prestretch-release, solvent swelling, compression, thermal, mold, and 3D printing. By optimizing fabricating parameters, it is possible to produce buckles with different morphologies and scale characteristics.

#### 2.3.1. Prestretch-Release

The mechanical prestretch-release method is the most widely used technique for fabricating buckled structures. Compared with other fabrication methods for producing buckled structures, the prestretch-release method features simple fabrication. It is compatible with a wide range of material systems and holds potential for scalable fabrication. Moreover, this method can be combined with other fabrication techniques. Typically, by imposing uniaxial or biaxial mechanical prestress on a flexible substrate of low modulus, followed by the deposition, lamination, or in situ growth of a rigid conductive layer of high modulus on its surface, and finally releasing the prestress, periodic buckles are developed on the conductive layer [15].

**Figure 3:**
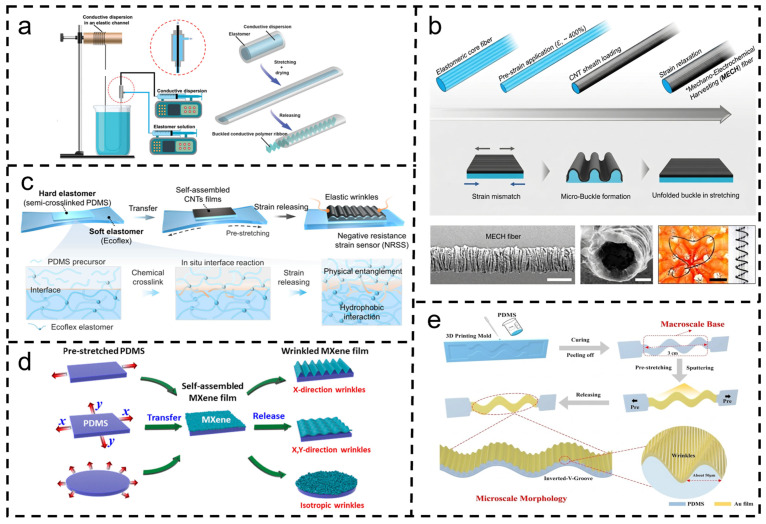
Buckled structure produced using a prestretch-release strategy. (**a**) A conductive strip with a corrugated morphology in a thermoplastic elastomer (TPE) channel. Reproduced with permission [67]. Copyright 2020 Wiley-VCH. (**b**) Manufacturing processes and morphologies of mechano-electrochemical harvesting (MECH) fibers. From left to right, the scale bars are 200 μm, 200 μm, and 1 cm. Reproduced with permission [31]. Copyright 2022 American Chemical Society. (**c**) A bio-inspired negative resistance strain sensor (NRSS) with a buckled structure. Reproduced with permission [27]. Copyright 2025 American Chemical Society. (**d**) Fabrication and structural characterization of buckled MXene films. Reproduced with permission [68]. Copyright 2023 American Chemical Society. (**e**) The design and fabrication process of the combinatorial bionic hierarchical (CBH) sensor, inspired by serpentine and butterfly wing structures, and the microstructure of the CBH sensor. Reproduced with permission [69]. Copyright 2024 American Chemical Society.

As shown in Figure 3a, the coaxial wet-spinning method was employed to continuously fabricate coaxial fibers composed of an aqueous solution of PEDOT/PSS/polyethylene-block-poly (ethylene glycol) (PBP) encapsulated in thermoplastic polyester [67]. Subsequently, different levels of prestress were applied to the fibers. When the inner wall solution cured, prestress was released, resulting in a conductive inner core with a buckled structure that was protected by an elastomeric outer shell. The coaxial buckled fibers produced not only exhibit good electrical resistance stability but also outstanding durability. Similarly, Sim et al. [31] pre-stretched the flexible fibers (Spandex) to a strain of 400% and then coated their surfaces with a film of MWCNTs. After releasing the prestress in the elastomer, a buckled structure spontaneously developed on the fiber surface. Young’s modulus of the fabricated fibers is as low as 2 MPa, which is similar to that of human skin and other tissues; therefore, it is suitable for the application in wearable energy devices (Figure 3b). In addition, by further increasing the prestress of the fiber substrate, buckled structures with multi-scale dimensional features can be fabricated. Liu et al. [32] wrapped oriented CNT films around a rubber fiber core prestretched to a strain of 1400%, and a multi-level buckled structure with alternating axial and circumferential buckles was achieved after releasing the prestress. The resulting flexible electrode exhibited a resistance change of less than 5% when subjected to tensile strains as high as 1000%.

Unlike fibers, films allow for the pre-straining of multiple directions. Among these, uniaxial pre-stretching is the most common method. As shown in Figure 3c, uniaxial prestretching was applied to the Ecoflex substrate, on which a layer of high-modulus semi-crosslinked CNT/PDMS film was deposited [27]. After releasing the prestress, a buckled structure formed on the film’s surface. The resulting buckled sensor exhibits a wide detection range, high sensitivity, and excellent durability. It is worth noting that the effects of magnitude and direction of prestress on morphology and functionality of the buckles are scarcely illustrated. Yang et al. [68] applied uniaxial, biaxial, and multi-directional pre-stretching to PDMS substrates, which were deposited with an MXene layer. After releasing the pre-strain, 1D, 2D, and isotropic buckled structures were obtained, respectively. The fabricated sensors, through biaxial and multi-directional stretching, can monitor deformations in multiple directions and provide a strategy for the development of multi-directional sensor devices. In addition, combining prestretch-release with other preparation methods, multi-level buckled structures on the membrane can also be fabricated. As shown in Figure 3e, inspired by a combination of serpentine and butterfly wing structures, Zong et al. [69] cast PDMS into a serpentine mold to produce a PDMS film with a macroscopic buckled structure. Subsequently, by depositing Au onto a pre-stretched PDMS film and then releasing it, a multi-level buckled structure was successfully fabricated. The flexible strain sensors developed based on this structure feature ultra-high sensitivity, wide detection range, and excellent response and durability.

The buckled structures of the prestretch-release method are influenced by factors such as substrate modulus, pre-strain degree, and interfacial adhesion state. This method is generally more suitable for planar or geometrically regular flexible substrates, and it is difficult to achieve uniform stress transfer on complex 3D surfaces or discontinuous surfaces. In addition, this method has weak control over the location, direction, and geometry of the buckles, making it difficult to fabricate precisely patterned buckled structures.

#### 2.3.2. Solvent Swelling

Examples of buckled structures formed by swelling can be found everywhere in daily life, such as fingers that have been soaked in water for a long time or dumpling wrappers that have cooled after being cooked. When polymers are immersed in solvent, solvent molecules are absorbed, causing the flexible polymer substrate to swell. Once solvent is removed, the substrate shrinks rapidly, but the conductive material on the surface remains unchanged throughout this process. Differences in the expansion ratios of different materials generate osmotic forces, which are then converted into in-plane compressive stresses, ultimately inducing the formation of buckled structures. Compared with the prestretch-release method, the solvent swelling method is suitable for non-spherical and complex curved substrates. In addition, this method can spontaneously form multi-scale, hierarchical buckled morphologies, thereby enabling the fabrication of the complex buckled structures. Kim et al. [70] placed UV-ozone-treated PDMS surfaces in various organic solvents, and they observe the formation of buckled structures on the surfaces. Moreover, they concluded that the wavelength and amplitude of the swelling-induced buckles were determined by the material’s geometric dimensions, modulus, and degree of swelling. Based on the aforementioned swelling theory, Vandeparre et al. [71] found that the morphology and periodicity of buckles can be controlled by constraining the swelling region. Gao et al. [19] placed a plasma-treated PDMS substrate in the organic solvent, causing the substrate surface to form a buckled structure. They then deposited a layer of conductive material onto the cured buckled surface to fabricate a highly sensitive pressure sensor. Lv et al. [72] swelled polyurethane (PU) fibers with tetrahydrofuran (THF) to create a buckled structure on their surface, and then AgNPs/MXene/CNTs were deposited. Finally, a fiber sensor with a wide detection range was developed with a long service life and broad operating range, which can be successfully applied in medical monitoring and human–computer interaction.

**Figure 4:**
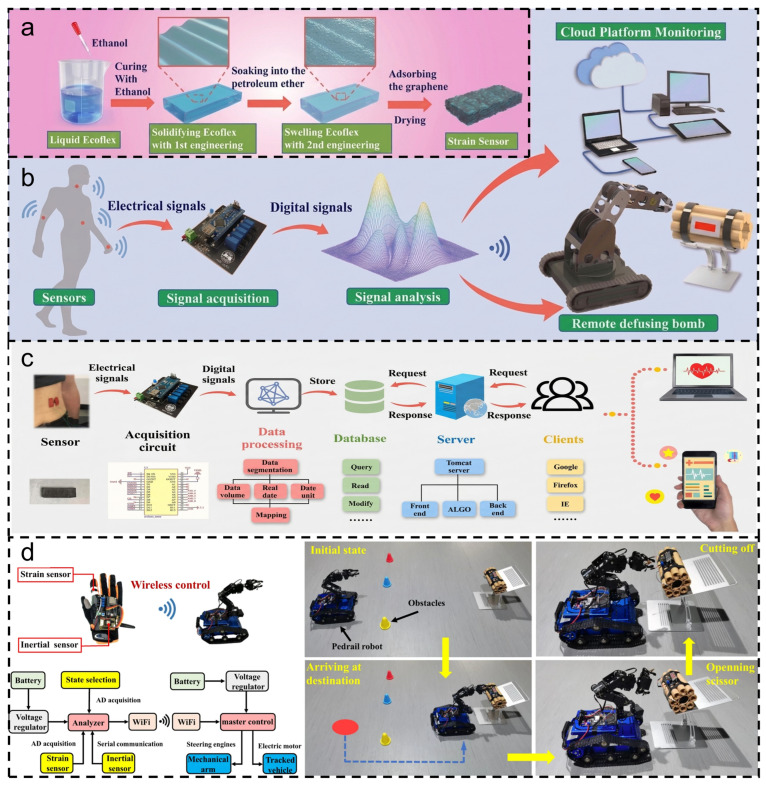
Buckles produced using the swelling method and their applications. (**a**) Schematic illustration of the preparation of the buckled structure and (**b**) its applications, including (**c**) big data cloud monitoring and (**d**) smart gloves and pedal robots. Reproduced with permission [73]. Copyright 2022 Nature Publishing Group.

It is worth noting that combining swelling with other methods can also produce multiscale buckled structures with varying dimensional characteristics. As shown in Figure 4a, Zhou et al. [73] first cured Ecoflex with ethanol and then formed wavy buckles on its surface by casting it over a template. And then it was immersed in petroleum ether to create even finer buckles on its surface. Finally, graphene is deposited onto the surface of the Ecoflex substrate, featuring a multi-level buckled structure, to fabricate a flexible strain sensor. When subjected to tensile deformation, the micro-buckles cause the conductive layers to separate abruptly and create a large number of internal voids, providing extremely high sensitivity. When strain increases, the larger nickels gradually participate in the deformation, ensuring that the circuit connection is maintained even upon large tensile stress. Based on the advantages of the aforementioned multi-level buckled structure, Zhou et al. [73] further applied the fabricated flexible strain sensors to the real-time detection and acquisition of human physiological signals. Meanwhile, signal acquisition, analysis units, and a cloud-based monitoring platform were established (Figure 4b,c). Furthermore, as shown in Figure 4d, such a strain sensor with a multi-level buckled structure can also be used in smart gloves to control robotic arms in real time for remote bomb disposal. In addition, Qu et al. [74] developed a gas sensor based on a swelling strategy. When a sensor is exposed to a volatile organic compound (VOC), the underlying elastomer absorbs gas molecules and begins to expand, forming a buckled structure on the surface. This causes a large number of conductive pathways on the surface to break, resulting in a sharp increase in contact resistance and generating a positive resistance change signal, whereby VOCs can be detected.

The solvent swelling method has stringent requirements for materials, and the flexible substrate used must undergo controllable volumetric expansion in the selected medium. This method typically relies on specific organic solvents, which may cause issues such as solvent residue and chemical contamination. In addition, the conventional swelling process is often isotropic, and the resulting compressive stress lacks clear directionality, making it difficult to precisely control the orientation, wavelength, and amplitude of buckled structures. The fabricated buckled morphology is also susceptible to factors such as swelling time, medium concentration, diffusion rate, temperature, and substrate thickness, leading to insufficient large-area structural uniformity and batch-to-batch reproducibility.

#### 2.3.3. Compression

Compression is another simple method of creating buckled structures, which can be applied to rigid conductive materials and force them to buckle and form buckles. The compression method allows active materials to be pre-folded before transfer. This facilitates the fabrication of highly conductive and transparent stretchable layers. Kim et al. [66] successfully produced transparent, stretchable conductors by applying biaxial compression on a network of AgNWs floating on water to create a buckled structure and then transferred such a buckled structure onto a PDMS substrate. Using this method, Kim et al. [75] further developed a strain sensor capable of detecting bidirectional strain and applied it to the detection of multidirectional strain of the elbow joint.

The compression method is typically applied to specific films, floating films, or nanomaterial networks. The processes of compression, transfer, and interfacial fixation are relatively complex and place high demands on processing conditions and operational precision. Therefore, the compression method still presents certain limitations in large-area uniform fabrication and scalable application.

#### 2.3.4. Thermal Treated

The thermal-treated method is similar to the previous methods. Conductive layers are attached to a thermally expanded substrate, and buckles are formed by the compressive stress during cooling. Compared with other buckled fabrication methods, the thermal-treated method is simpler and more cost-effective. This method utilizes the thermal expansion mismatch between layers or the shrinkage characteristics of shape memory polymers, enabling rapid formation of buckled structures by adjusting the temperature. Park et al. [20] uniformly sprayed a solution of SWCNTs onto a PS film. When the PS was heated past its glass transition temperature, the PS shrunk, causing the CNT thin film to buckle and form highly self-similar buckled structures. In addition, by controlling the boundaries of PS film shrinkage, buckles with unidirectional (1D) or bidirectional (2D) morphologies can be developed.

The thermal-treated method typically has specific requirements for the thermal response characteristics, heat resistance, and dimensional stability of the substrate material. The thermal treatment may also introduce residual stress in the flexible substrate or functional layer and induce interfacial damage, structural deformation, or degradation of material properties, thereby affecting the stability of the device.

#### 2.3.5. Mold

Although the aforementioned preparation method offers the advantages of simplicity and efficiency, it is often difficult to accurately construct buckled structures with specific shapes. The mold method produces a substrate with a predetermined corrugated surface by pouring the substrate material solution into a specially designed mold. Compared with other methods, the mold method enables customized design of buckled structure morphology by adjusting the geometry and characteristic dimensions of the mold and offers high dimensional control precision and structural repeatability. Therefore, this method is suitable for fabricating regular buckled structures with specific morphologies and precise dimensional requirements. Wang et al. [76] used compact discs (CD) as molds and employed a hot-pressing method to replicate the structure of the discs on the surface of PET film. Then, by imprinting tellurium (Te) nanowires synthesized via liquid-phase synthesis onto a PET substrate, flexible electronic devices with a concentric, periodically rippled structure were obtained. As shown in Figure 2e, inspired by corrugated cardboard structures, Li et al. [21] utilized a templated molding method to fabricate a multi-layered buckled structure from PDMS/MWCNT composites. Of note, stress concentration upon pressure can be effectively avoided through unfolding. The resulting pressure sensor demonstrated a linear response across the 0.002–500 kPa range. In addition, Meena et al. [77] utilized a coupled transfer-printed-spin coating technique. Polyvinylidene fluoride (PVDF) was spin-coated onto a wrinkled PDMS mold, annealed, and subsequently separated from the mold to transfer-print an inverse replica microstructure onto the freestanding PVDF film. The hybrid piezoelectric-triboelectric nanogenerator based on this imprinted buckled structure exhibits high tactile sensitivity and a broad force sensing range.

The buckled patterns and dimensions fabricated by the mold method depend on the mold, and the fabrication process has relatively low scalability. The cost of mold fabrication is high, and during the peeling process, the surfaces of certain special buckled structures are prone to deformation.

#### 2.3.6. 3D Printing

Previously proposed methods to fabricate buckles are generally not suitable for constructing 3D buckled structures. Furthermore, it is difficult to control the precise shape and size of buckles. However, with the development of additive manufacturing technology, buckles can be fabricated by using 3D printing technology. Unlike the above-mentioned fabrication methods that rely on spontaneous instability or in-plane deformation, 3D printing can directly control the characteristic dimensions and spatial morphology of buckled structures through digital modeling and achieve the fabrication of customized 3D buckled structures. Therefore, this method offers high flexibility in 3D geometric design and personalized manufacturing. Wang et al. [22] used PµSL to print a base polymer with a buckled structure, then coated the printed microstructures with thin Au films. The resulting flexible electrodes exhibit high stretchability and a low resistance change rate. This promotes the application of FEDB in the fields of flexible electrodes, strain and pressure sensors, energy, and electrothermal devices.

However, the available materials of 3D printing have a limited range, and existing printing materials generally struggle to simultaneously meet the requirements of high flexibility, good electrical conductivity, printability, and long-term stability. In addition, 3D printing still has certain limitations in terms of manufacturing speed and processing resolution.

## 3. Applications of FEDB

In view of the unique characteristics of buckled structure, the fabricated electronic devices are able to maintain a stable conductive network even under large deformations. Additionally, the presence of buckles can increase the specific surface area of functional materials. Meanwhile, it can also serve as a microstructure that provides additional functions, such as hydrophobicity. In recent years, its application has been gradually expanded to encompass emerging application scenarios such as healthcare, health monitoring, human–computer interfaces, and underwater monitoring. Table 2 shows the application scenarios described above and their corresponding performance requirements.

### 3.1. Flexible Electrodes

**Figure 5:**
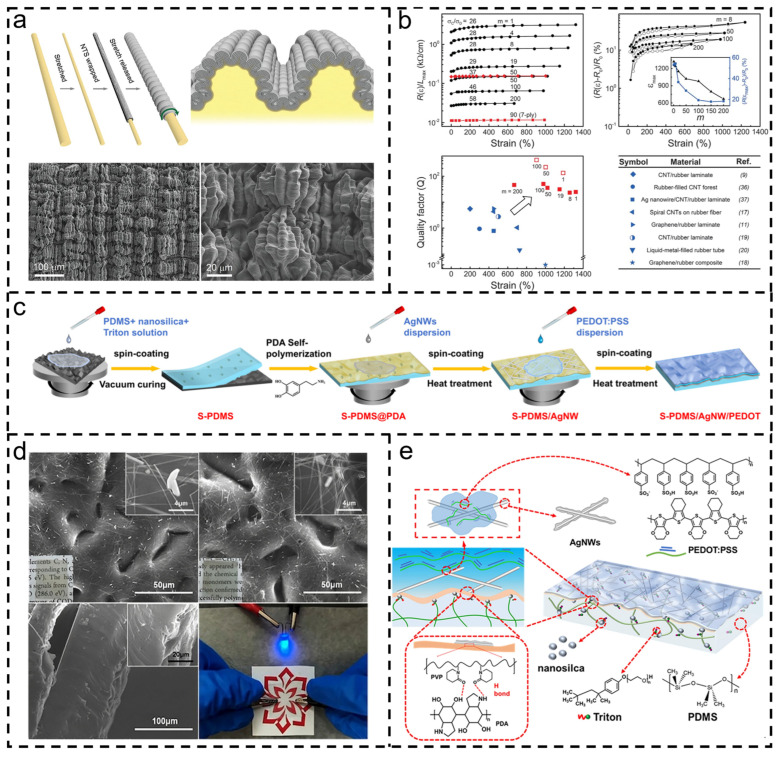
Application of buckled structures in flexible electrodes. (**a**) The fabrication process and morphology of skin-core fibers with a layered buckled structure. (**b**) Strain dependence of the electrical properties of core-sheath fibers. (**c**) The preparation process of soft PDMS(S-PDMS)/AgNW/PEDOT composite films. (**d**) SEM image of a cross-section of S-PDMS/AgNW/PEDOT and a photograph of the illuminated LED. (**e**) Bonding mechanism at the S-PDMS/AgNW/PEDOT interface. (**a**,**b**) Reproduced with permission [32]. Copyright 2015 American Association for the Advancement of Science. (**c**–**e**) Reproduced with permission [28]. Copyright 2025 American Chemical Society.

Generally, metal electrodes have high electrical conductivity but low stretchability, whereas organic electrodes have high stretchability but lower electrical conductivity than metal electrodes [78,79]. By incorporating a buckled structure and attaching a rigid conductive layer to a flexible substrate with high stretchability, it is possible to achieve both high stretchability and high conductivity. Upon stretching, the buckled structures could absorb strain by unfolding, which inhibits stress concentration and microcrack propagation within the conductive layer and maintains the stability of the conductive path. The wavelength and amplitude of the buckles determine the electrode’s deformability. Generally, a larger amplitude and a shorter wavelength provide the conductive network with more strain buffer space, thereby enabling a higher stretch limit. Table 3 provides detailed information on the flexible electrodes fabricated based on buckled structures. As shown in Figure 5a, Liu et al. [32] used a prestretch-release method to fabricate highly stretchable sheath-core conducting fibers created by wrapping carbon nanotube sheets oriented in the fiber direction on stretched rubber fiber cores. The prepared conductive fibers exhibit an extremely high-quality factor (Q). The resulting structure exhibited distinct short- and long-period sheath buckling that occurred reversibly out of phase in the axial and belt directions, enabling a resistance change of less than 5% for a 1000% stretch (Figure 5b). In addition, an optical fiber capacitor was successfully fabricated based on this structure. Under a tensile strain of 950%, the capacitance increased by 860%. Based on the principles of fractal geometry, a stretchable conductive fiber with hierarchical buckles inspired by the unique shape of the maple leaf was fabricated by combining surface modification, interfacial polymerization, and improved pre-strain finishing methods [80]. Such hierarchically buckled conductive fiber (HWCF) not only exhibits excellent conductivity and strain insensitivity but also demonstrates long-term durability (>1000 stretch-release cycles). It can be applied as highly stretchable electrical circuits for illumination and monitors for the human motion under large strains through tiny and rapid resistance changes as well. Furthermore, flexible electrodes are expected to have higher transparency. As shown in Figure 5c–e, a fluor-silane-modified sandpaper template was employed to create a nano-silica-enhanced PDMS composite membrane with a buckled surface structure [28]. Through spin-coating and thermal treatment, AgNWs and PEDOT: PSS were sequentially deposited onto the structured membrane, yielding a dense, well-adhered conductive network. The resulting flexible composite electrode not only retained optical transparency but also exhibited high tensile strength (3.2 ± 0.4 MPa; elongation at break, 210 ± 20%) and low hysteresis (hysteresis loss of only 0.0567 after 100 cycles at 50% strain).

**Figure 6:**
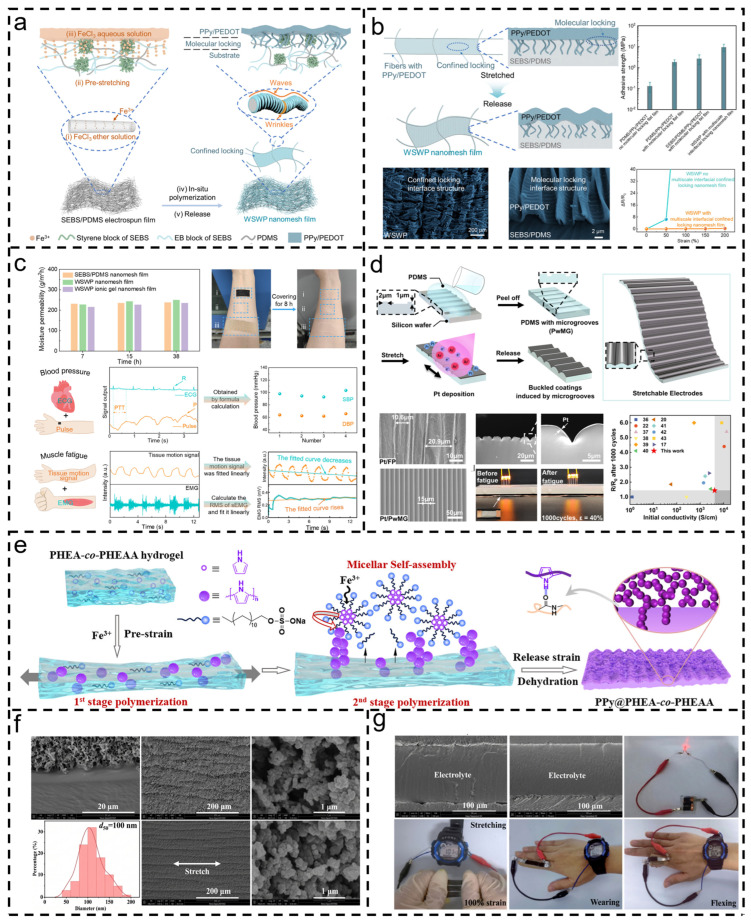
Application of buckled structures in flexible electrodes. (**a**) Schematic illustration of the preparation process for wavy SEBS/PDMS and PPy/PEDOT (WSWP) and their multi-scale interface-constrained locked structures. (**b**) Scanning electron microscope images of the multi-scale interfacial confinement mechanism of WSWP. (**c**) Applications of WSWP nanomembranes in human blood pressure and muscle fatigue. (**d**) Schematic diagram of the fabrication process and morphology of the Pt-PDMS with microgroove electrodes. (**e**) Schematic illustration of the surfactant-assisted preparation process for PPy@PHEA-co-PHEAA. (**f**) SEM image of PPy@PHEA-co-PHEAA in the unstretched state and after stretching to 100% strain. (**g**) Scanning electron microscope (SEM) image of SSC_PPy_ and an optical image of its application. (**a**–**c**) Reproduced with permission [29]. Copyright 2025 Wiley-VCH. (**d**) Reproduced with permission [82]. Copyright 2025 American Chemical Society. (**e**–**g**) Reproduced with permission [83]. Copyright 2025 American Chemical Society.

Stable electrical conductivity is crucial for flexible electrodes. However, the poor adhesion between active conductive material and flexible substrate often leads to instability in electrical conductivity during long-term operation. In view of this, Liang et al. [29] proposed a multiscale interface confinement strategy. As shown in Figure 6a,b, this strategy combined molecular entanglement between the conductive polymer and the substrate with physical confinement in the electrospun membrane pores to construct a PPY/PEDOT conductive layer with a buckled structure on a SEBS/PDMS substrate. Such a flexible electrode has high interfacial adhesion strength (9.48 MPa). Figure 6c shows that this flexible electrode exhibits positive responses in applications for monitoring blood pressure and muscle fatigue. Huang et al. [82] utilized a silicon mold to pattern microgrooves onto a PDMS substrate, followed by the deposition of a platinum nanofilm during a pre-stretched state. When the pre-strain was released, the localized difference in stiffness at the microgrooves guided the structural buckling, resulting in a controlled, micro-convex stripe morphology. (Figure 6d). Compared with the conventional metal film electrode with a randomly wavy shape, the micro-convex stripe-shaped platinum nanofilm significantly suppresses the strain concentration and the crack propagation of the nanofilm during the stretch−release cycles. The resulting electrode maintains the high electrical conductivity (4.1 × 10^5^ S/m) of traditional metal membrane electrodes with random wavy shapes and also exhibits excellent resistance stability and durability. The resistance after 1000 cycles is half that of conventional electrodes. Furthermore, as shown in Figure 6f, Yang et al. [83] firmly anchored porous PPy onto a flexible PHEA-co-PHEAA substrate to fabricate a stretchable electrode with a buckled surface structure. This electrode could power a light-emitting diode (LED) lamp or an electronic watch (Figure 6g).

### 3.2. Flexible Strain Sensors

**Figure 7:**
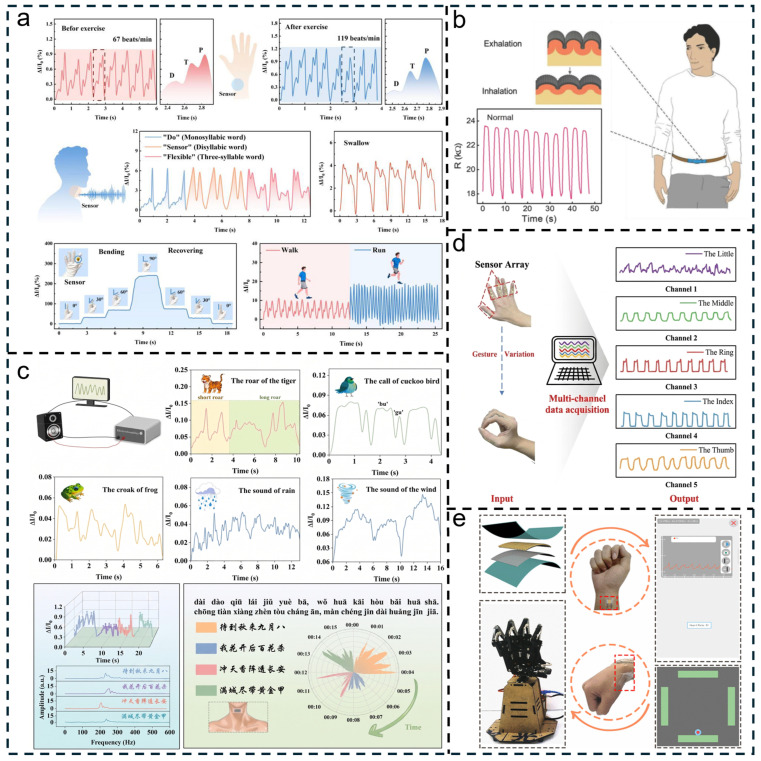
Application of buckled structures in wearable flexible strain sensors. (**a**) For monitoring human pulse, voice, swallowing, and movement. Reproduced with permission [21] Copyright 2025, Wiley-VCH. (**b**) For detecting human respiration. Reproduced with permission [84] Copyright 2017, Wiley-VCH. (**c**) For monitoring human and animal sounds. The English translation of the non-English letters is: “When autumn comes, on the eighth day of the ninth month, / My flowers bloom, all others fade. / Their fragrance soars through Chang’an, / The whole city is clad in golden armor.” Reproduced with permission [85] Copyright 2025, Elsevier. Used for (**d**) gesture recognition and (**e**) human–computer interaction. (**d**) Reproduced with permission [69] Copyright 2024, American Chemical Society. (**e**) Reproduced with permission [86] Copyright 2023, American Chemical Society.

Flexible strain sensors are electronic devices that convert mechanical deformations (such as tension, compression, bending, and torsion) into electrical signals. Such sensors have a wide range of applications in fields including biomedicine, smart health monitoring, human–computer interaction, and soft robotics. Under stress, the continuous unfolding of the buckled structure accommodates the applied strain, which expands the detectable strain range and enhances the linearity of the sensor’s response. Furthermore, the specific wavelength and amplitude of the buckles are correlated with the extent of the strain range and the degree of linearity. Generally, larger amplitudes and appropriate wavelengths provide a greater structural cushion, enabling broader stretchability and a more stable, linear resistance change during deformation. Table 4 provides detailed information on the flexible strain sensors fabricated using a buckled structure. As shown in Figure 7a, Li et al. [21] developed a wearable flexible strain sensor composed of PDMS and MWCNTs, which can detect wrist pulse before and after physical activity, laryngeal vibrations during speech, swallowing movements, finger bending, and walking and running states. Moreover, Wang et al. [84] designed a strain sensor composed of CNT sheets and rubber fibers featuring high linearity, fast response time, high resolution, and excellent stability and can successfully detect human respiration (Figure 7b). As shown in Figure 7c, Tao et al. [85] fabricated a flexible strain sensor based on PET fabric and a conductive layer of MWCNTs. This sensor features a wide pressure measurement range (0–100 kPa), high sensitivity (1239 kPa^−1^), a low detection limit (5 Pa), and long-term durability (over 4000 cycles). By analyzing the frequency responses, the sensor excels in detecting sound signals, including environmental sounds (e.g., animal calls and wind noises) and human speech, which further expands the application scenarios for strain sensors. When the range of applications expands, the detection accuracy of strain sensors has also improved. Inspired by a combination of serpentine and butterfly wing structures, Zong et al. [69] developed a CBH flexible strain sensor featuring a multi-layered buckled structure, which not only has a high strain range of 150% but also boasts a gauge factor (GF) as high as 2416.67. Such a CBH sensor array can detect different bending angles at the finger joints, wrist, and elbow and can be used for the precise recognition of sign language gestures, with the potential to enable seamless communication between sign language users and others (Figure 7d). In addition, Zhang et al. [86] fabricated a tunable buckle clamp down structure (WCDS) crack strain sensor based on high Poisson’s ratio material with high sensitivity, high stability, and a wide strain range. This sensor enables recognition of hand gestures and game control (Figure 7e), and it also has the potential to be used in home health monitoring and human−machine interaction.

**Figure 8:**
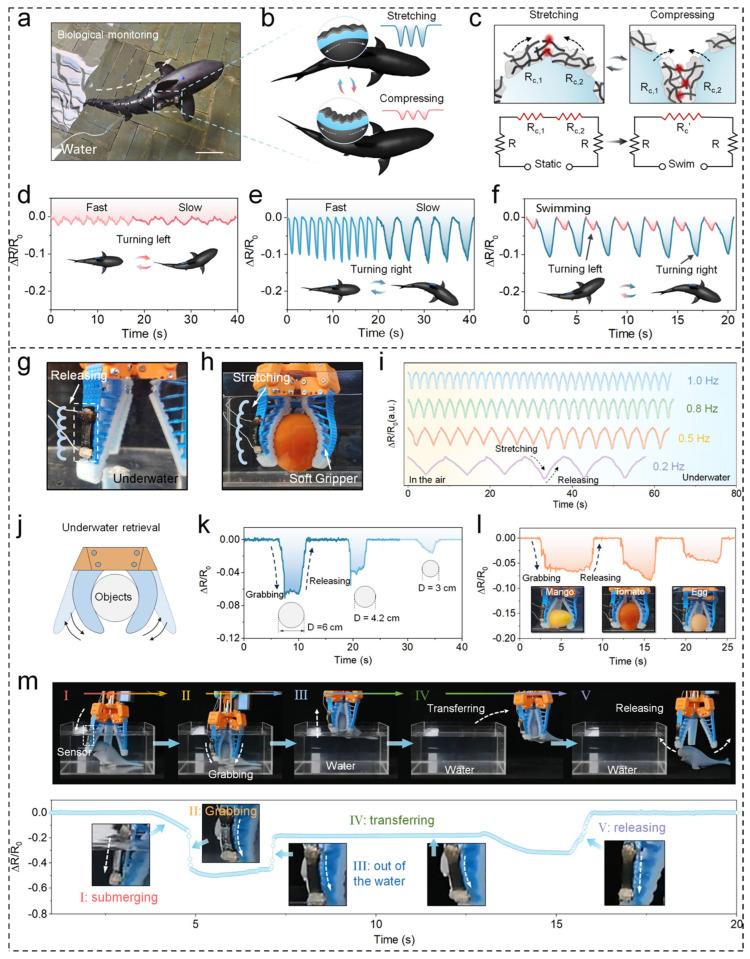
Applications of buckled structures as flexible strain sensors underwater, monitoring fish movement and identifying objects grasped underwater. (**a**) Photograph of NRSS for detecting underwater fish movement, where the sensors are integrated into the tail of the fish (scale bar: 10 cm). (**b**) Schematic illustration of two movement patterns of fish, including turning left (compressing state) and turning right (stretching state). (**c**) Mechanism diagram of the two motion modes in NRSS. Relative resistance curves of fish (**d**) swimming left and (**e**) right, showing a small signal when swimming left and a large signal when swimming right. (**f**) Real-time resistance curve of fish swimming forward. (**g**,**h**) Photos of the NRSS gripper releasing and grabbing underwater. (**i**) Relative resistance curves of the NRSS gripper under different frequencies, operating stably underwater and in air. (**j**) Schematic diagram of underwater retrieval. (**k**) Relative resistance variation of NRSS for size detection of underwater objects. (**l**) Relative resistance variation of the NRSS gripper for underwater object recognition. (**m**) Photos of NRSS-integrated flexible gripper during the grabbing process (scale bar: 10 cm) and the corresponding relative resistance time curve, including the stages of entry, grab, exit, transfer, and release. Reproduced with permission [27]. Copyright 2025 American Chemical Society.

Nowadays, flexible strain sensors have reached maturity in the conventional applications mentioned above and are beginning to expand into emerging application areas such as underwater sensing. Li et al. [27] applied a conductive layer of CNTs to Ecoflex and PDMS buckles, successfully fabricating hydrophobic flexible strain sensors. As shown in Figure 8a, the superhydrophobic NRSS was integrated into the tail of the shark model, and the motion was further monitored. During this process, shark left turns (NRSS is compressed) and right turns (NRSS is stretched) can be accurately identified and detected (Figure 8b). The motion pattern mechanism is analyzed in Figure 8c. When the shark turned to the right, the NRSS was stretched, and the CNT clusters at the top of the 3D buckles contacted each other, causing a reduced resistance. Owing to the substantial dynamic contact area and high strain at the top, a more remarkable negative resistance change can be obtained. Similarly, when the NRSS was compressed, CNT clusters at the bottom of the buckle also contacted, resulting in a reduction in resistance. As shown in Figure 8d, e, when the biomimetic fish turns left (compression), it outputs a weak, low-amplitude signal. When it turns right (stretching), it outputs a strong, high-amplitude signal. Furthermore, the sensors are capable of accurately detecting the different swimming frequencies of the biomimetic fish. As shown in Figure 8f, the sensor can accurately record signals while the bionic fish swims continuously forward. The NRSS is also integrated into a three-jaw flexible gripper for underwater object recognition and retrieval. As shown in Figure 8g,h, the typical superhydrophobic underwater characteristics of the NRSS can be clearly observed. The NRSS was stretched during grabbing, and there is a remarkable negative resistance change. Moreover, NRSS presented excellent electrical stability in both air and underwater environments and exhibited a sensitive electrical signal response to grab frequencies (Figure 8i). As shown in Figure 8j–l, the size of the object being grasped underwater can be determined by changes in the resistance feedback. As a demonstration, the complete process of underwater grabbing can be monitored in real time, including the stages of entry, grab, exit, transfer, and release (Figure 8m).

### 3.3. Flexible Pressure Sensors

**Figure 9:**
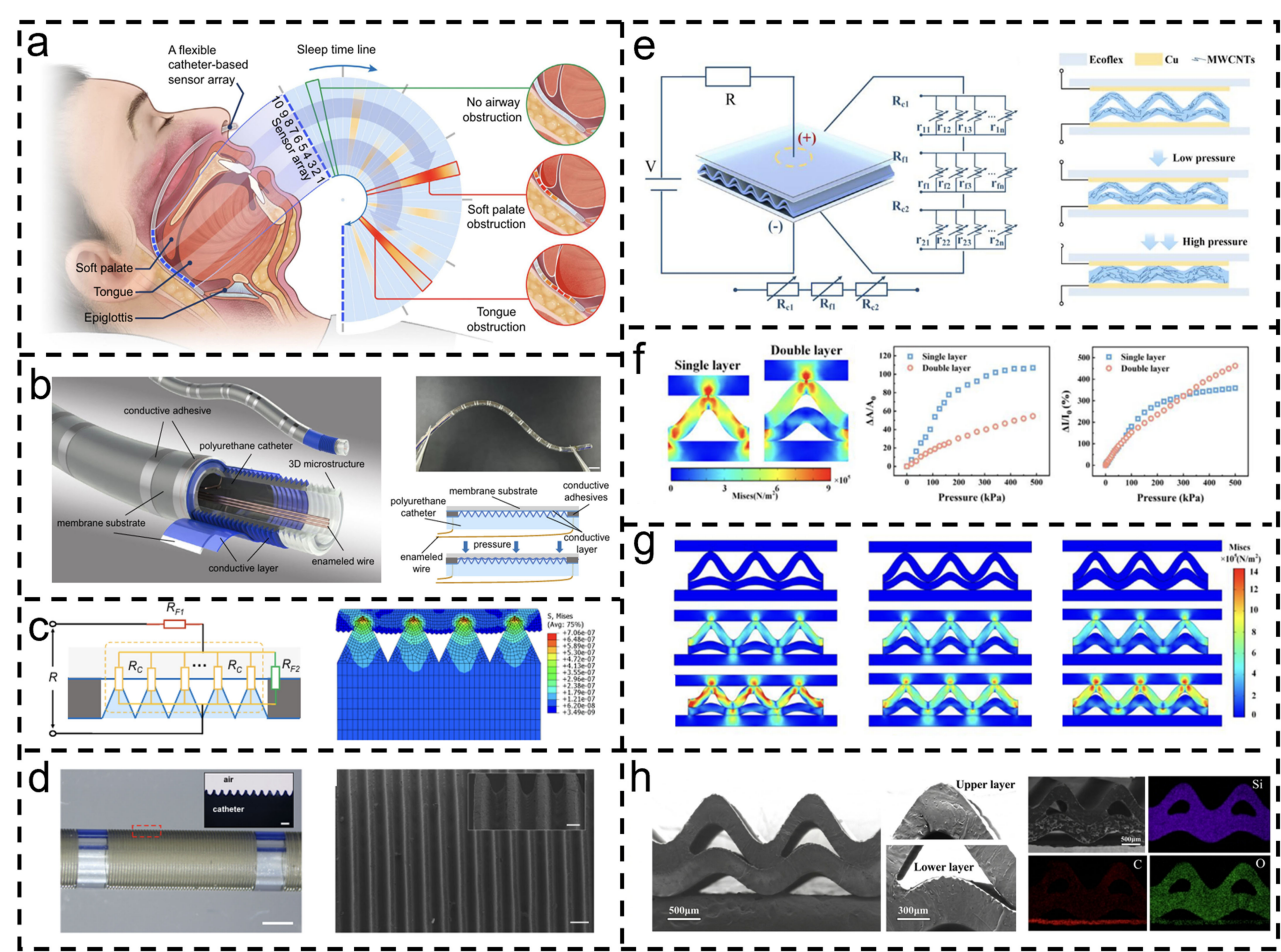
Applications and mechanisms of buckled structures in pressure sensors. (**a**) Schematic diagram of a sensor array on a flexible catheter for OSA monitoring. (**b**) Schematic diagram of a multi-layer flexible sensor array, photograph of a sensor array based on a flexible catheter, and schematic diagram of a single sensing unit. Scale bar = 5 mm. (**c**) Simplified electrical model of a flexible piezoresistive sensor (left) and simulation of flexible deformation at the interface between the piezoresistive sensor microstructure and the substrate (right). (**d**) Photograph of the catheter after etching the microstructure on the catheter wall and a magnified view of the microstructure under a metallurgical microscope (left). The outer scale bar is 5 mm and the inner scale bar is 100 μm. SEM image of the microstructure on the catheter surface (right). Scale bar = 100 μm. (**e**) Sensing mechanism of the double-layer buckled structure flexible pressure sensor (DCPS). (**f**) Simulations of stress distribution (left) and contact area (center) for single-layer and double-layer buckled structures under the same pressure. Current response curves of the piezoresistive sensing layers of single-layer and double-layer corrugated structures under pressure (right). (**g**) Stress distribution of double-layer corrugated structures with different height ratios (upper-to-lower layers 4:1, 2:1, 4:3) under the same pressure. (**h**) SEM cross-section of the DCPS (left) and elemental distribution map of Si, C, and O in the cross-section of the double-layer corrugated structure (right). (**a**–**d**) Reproduced with permission [25]. Copyright 2024 Springer Nature. (**e**–**h**) Reproduced with permission [21]. Copyright 2025 Wiley-VCH.

The buckled structure formed on the conductive layer stabilizes the conductive pathway under conditions of large deformation, serving as a functional microstructural unit for pressure sensors. When external pressure is applied, deformation of these units results in variations in the effective contact area, thereby contributing to the sensor’s sensitivity. The wavelength and amplitude of the buckles also determine the geometric dimensions of these microstructural units. The dimensions of these units govern their structural compressibility and the rate at which the contact area changes during compression, thereby modulating the pressure-sensing performance. As shown in Figure 9a, Shang et al. [25] developed a tubular array pressure sensor with a regular buckled surface. By inserting the tube into the upper airway of the human nasal cavity, it enables the monitoring of obstructive sleep apnea (OSA). As shown in Figure 9b–d, this catheter-based flexible pressure sensor array uses a PU nasogastric tube as its substrate, with 3D microstructures directly etched onto its surface using a femtosecond laser. The superior flexibility and small diameter of the catheter allow this sensor array to adapt to the angles of the upper respiratory tract, while the incorporation of a buckled structure enhances the sensitivity of pressure sensing. These features make the catheter a promising tool for accurately monitoring the human airway, providing strong support for addressing global health issues such as obstructive sleep apnea syndrome. Inspired by the corrugated paper structure, Li et al. [21] created a flexible piezoresistive sensor with a double-layer corrugated structure, fabricated from a PDMS/MWCNTs composite. When subjected to lower pressure, the buckles with greater amplitude come into contact with the electrodes first. When pressure increases, these buckles gradually flatten, while the structure of those with lower amplitude remains unchanged. With further increasing pressure, the buckles with greater amplitude reach their limit of extension, while the buckles with smaller amplitude gradually stretch out until the double-layer buckles are flattened. The sensor exhibits a sensitivity of 1.7 kPa^−1^ and linear response (R^2^ = 0.998).

**Figure 10:**
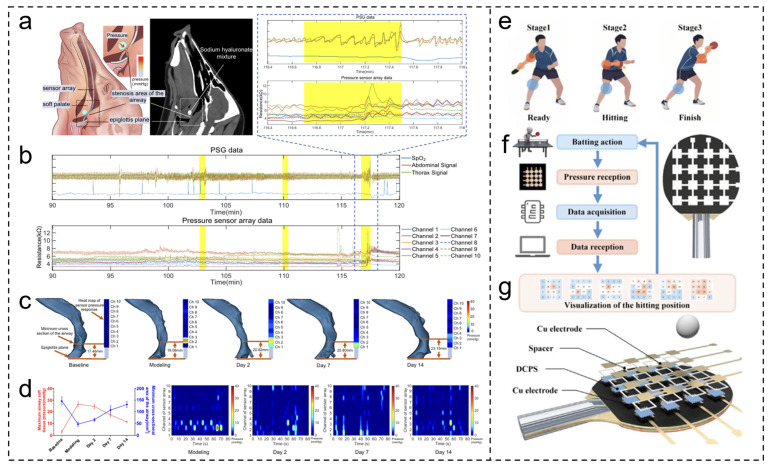
Applications of pressure sensors based on buckled structures. (**a**) Schematic illustration of a pressure sensor array based on a flexible catheter, demonstrated in vivo in a pig model of OSA. (**b**) Graphs showing the relationship between motion signals, abdominal motion signals, and blood oxygen saturation over time (bottom) and an enlarged inset of a representative OSA event (top). (**c**) Locations of the minimum cross-sectional area in airway CT images at different time points before and after modeling, and pressure responses measured by the sensor array during OSA events. (**d**) Representation of the minimum cross-sectional area of the upper airway and maximum airway soft tissue pressure during OSA events at different time points before and after modeling in the OSA pig model (left). Heatmap of obstruction pressure distribution during OSA events at different time points (right). (**e**) Schematic illustration of the relative current response of DCPS attached to an athlete during a stroke. (**f**) Flowchart of table tennis training guidance using a DCPS array. (**g**) Schematic of a DCPS array embedded in a table tennis bat for training guidance. (**a**–**d**) Reproduced with permission [25]. Copyright 2024 Springer Nature. (**e**–**g**) Reproduced with permission [21]. Copyright 2025 Wiley-VCH.

Figure 10a shows the catheter pressure sensor developed by Shang et al. [25]. It can be inserted into the airway of an OSA pig model for respiratory monitoring. During a sleep period lasting 90 to 120 min, the catheter pressure sensor monitored signals relating to chest movement, abdominal movement, and blood oxygen saturation. Furthermore, OSA events were successfully detected (Figure 10b). Furthermore, as shown in Figure 10c, a comparison was made between the anatomical location of the ‘minimum airway cross-section’ in the CT image and the ‘heat map’ of pressure distribution measured by the sensor array at the same time an OSA event occurred. It was found that the areas of high pressure identified by the catheter pressure sensors corresponded closely with the anatomically narrowest points of the airway shown in the CT image (with an error of only a few millimeters). Moreover, pressure sensors can perform continuous monitoring and quantitatively reflect the severity of the disease (Figure 10d). As shown in Figure 10e–g, Li et al. [21] used the DCPS developed for detecting ping-pong ball strike locations and correcting posture. To analyze the distribution of impact points more clearly, the sensor array was affixed to the surface of the table tennis bat. By integrating the data acquisition module with the transmission module, the group has successfully achieved real-time monitoring and statistical analysis of the ball’s landing point. By presenting shot-impact data in the form of a heatmap, the DCPS system not only helps athletes understand their own hitting habits but also provides coaches with objective data, enabling them to optimize training strategies accurately. In addition, Chang et al. [92] used a prestretch-release method to fabricate a conductive reduced graphene oxide (rGO) layer with a buckled structure on a PS substrate. The resulting pressure sensor exhibits high stretchability, high sensitivity, and a distinguishable pressure response. This buckled pressure sensor is used in the manufacture of robots capable of performing continuous surgical procedures. Specifically, such a pressure sensor can detect the robot−tissue contacts under joint stretches in real time to enhance the surgeon’s awareness of collision avoidance.

### 3.4. Flexible Energy Devices

Flexible energy devices based on buckled structure technology have attracted considerable attention due to their widespread application in wearable electronics. Energy devices can harvest and store energy from human movement, mechanical action, and solar radiation. The main types of energy devices are piezoelectric nanogenerators (PENGs), triboelectric nanogenerators (TENGs), and capacitors.

#### 3.4.1. PENGs

The generation of piezoelectricity primarily depends on the relative displacement of positive and negative charge centers and the resulting dipole moment that occurs when a piezoelectric material deforms under external mechanical stress. These mechanically induced polarized charges create an internal electric field and a potential difference at the two ends of material, which, in turn, drives the directed movement of free electrons in the external circuit, converting mechanical energy into electrical energy for storage. When undergoing dynamic movements such as expansion and contraction, the buckled structure generates continuous mechanical deformation through its own unfolding and folding, thereby inducing polarized charges and electrical signals within the piezoelectric material. The wavelength and amplitude of the buckles determine the efficiency of local strain transfer in piezoelectric materials under specific mechanical stimuli. A shorter wavelength typically increases the number of deformation nodes per unit area, thereby improving the device’s electromechanical conversion efficiency. To address the mechanical mismatch between rigid materials and soft matrices, Qi et al. [93] fabricated buckled lead zirconate titanate (PZT) ribbons on elastomeric substrates. This wavy geometry accommodates applied strain through structural unfolding rather than material stretching, thereby imparting stretchability to the inherently brittle PZT. Further, local probing of the buckled ribbons reveals an enhancement in the piezoelectric effect up to 70%. However, the buckled structures have limitations when applied to piezoelectric devices due to the uneven distribution of internal strain during deformation. To resolve this issue, Yea et al. [94] introduced a curvature-specific coupling electrode design for stretchable 3D inorganic piezoelectric nanogenerators. By utilizing patterned top and bottom electrodes to selectively collect charges from either the convex or concave regions, this design prevents charge cancellation and maintains stable energy harvesting performance under tensile strain. Sim et al. [31] fabricated MWCNT films with a buckled structure on B fibers. The resulting mechano-electrochemical harvesting (MECH) fibers exhibit low Young’s modulus and reversible flexibility. The researchers sutured the fibers directly into the stomachs and bladders of animal models and found that the fibers exhibited good biocompatibility. As the organs expanded or contracted, the buckles on the fibers’ surface could detect their dynamic movements by generating electrical signals as they unbuckled and closed.

#### 3.4.2. TENGs

TENG is a device that converts mechanical energy into electrical energy based on the coupling of the triboelectric effect and electrostatic induction. When two friction layers made of different materials come into contact and then separate, surface charges are generated due to differences in electron affinity. These charges create a potential difference between the electrodes through electrostatic induction, driving the flow of electrons and generating an electric current. TENG devices demonstrate exceptional performance in harvesting low-frequency, irregular interfacial friction energy generated by contact and sliding. The buckles not only provide the friction layer with a larger specific surface area to promote charge accumulation but also enhance the electrostatic induction effect during mechanical contact and separation, thereby significantly improving the electrical output performance of the triboelectric generator. The effective contact area of a triboelectric generator is closely related to the structural parameters of the buckles. Larger amplitudes and appropriate wavelengths can significantly increase the roughness of the friction surfaces, thereby generating higher surface charge densities and mixed-voltage outputs in contact mode. In the design of TENGs, introducing buckled structures effectively enlarges the contact area at the triboelectric interface. For instance, Song et al. [95] fabricated micro-wrinkled structures on polydimethylsiloxane (PDMS) surfaces using plasma treatment to serve as the triboelectric layer. Compared to flat elastomer surfaces, these micro-scale wrinkles increased the number of effective friction contact sites, thereby improving the efficiency of converting ambient mechanical vibrations into electricity for integrated self-charging power units. Furthermore, Xiao et al. [96] developed a TENG device based on a micro-crack-assisted wrinkled PEDOT: PSS film. The wrinkled morphology accommodated tensile strain, preventing film rupture during deformation, which allowed the device to independently distinguish between tensile strain and normal pressure without signal crosstalk. Meena et al. [77] fabricated a ferroelectric polyvinylidene fluoride (PVDF) polymer matrix. Folding the polymer can promote charge accumulation. Compared with conventional TENGs, the hybrid nanogenerators produced using this method not only offer higher hybrid voltage output and power density but also have a wider detection range.

#### 3.4.3. Other

In addition, Jang et al. [97] created a hierarchical buckled structure within the internal skeleton of a 3D PDMS polymer foam and subsequently coated the surface with MoS_2_ to impart conductivity. Supercapacitors produced using this method exhibit a specific surface area that is more than 60% greater and an energy storage capacity that is 8.3 times higher. It is worth noting that when subjected to a 50% tensile strain, the buckles can absorb the mechanical stress by flattening out, thereby maintaining the capacitor’s energy storage performance.

### 3.5. Other Applications

**Figure 11:**
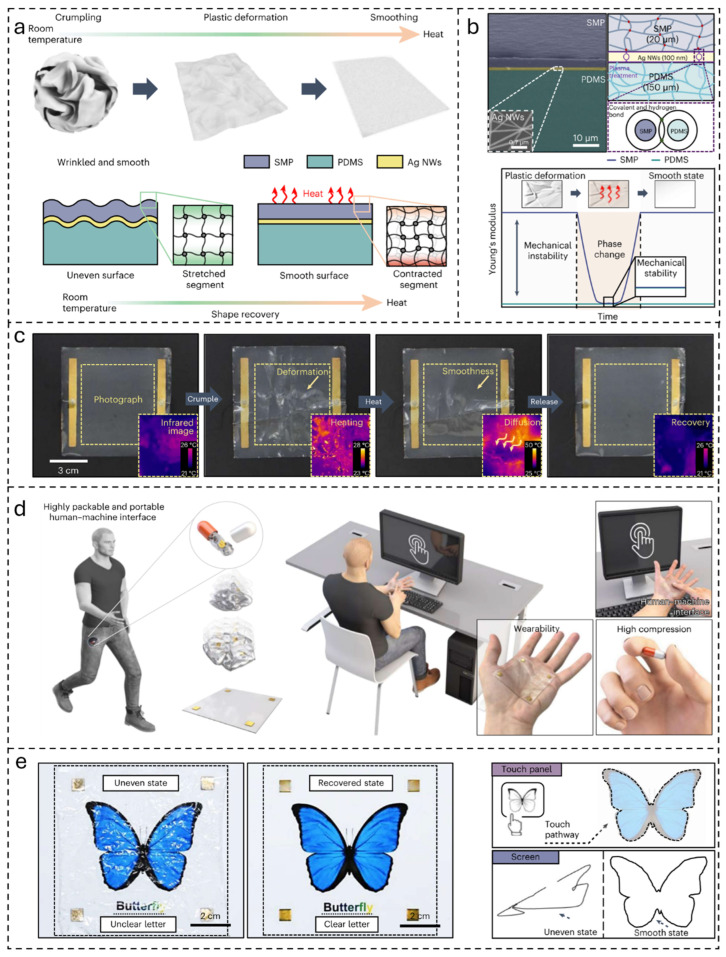
Applications of buckled structures in smart flexible touch panels. (**a**) The mechanism of buckle removal and smoothing caused by the relaxation of stretched polymer segments due to the thermal effect generated by AgNWs. (**b**) SEM image and schematic diagram of the covalently bonded HIS-layered structure and a schematic diagram illustrating the mechanical instability between SMP and PDMS caused by the phase transition of the SMP. (**c**) Photograph and infrared (IR) image of the recovery process of the plastically deformed HIS. (**d**) Schematic diagram of the buckled structure used in a human–machine interface. (**e**) The surface of the touch panel in both uneven and flat conditions, and the results of handwritten content written on the touch panel based on its state. (**a**–**e**) Reproduced with permission [26]. Copyright 2023 Springer Nature.

In addition to the aforementioned applications, buckled structures have other applications such as communication antennas, haptic interaction systems, and electrothermal devices. As shown in Figure 11a, inspired by the surface topography of butterfly wings, Roh et al. [26] combined SMP, AgNWs, and PDMS to fabricate a heterogeneously integrated structure (HIS) device. As shown in Figure 11b, through thermal treatment alone, the HIS device can smooth out the buckles formed during external crumpling and pressing, recovering its original smooth surface. The continuous optical and infrared images illustrate the trends in stiffness and surface topography at different temperatures. When a voltage is applied to HIS, current flows through the low-resistance regions, producing only slight ripples. The Joule heating generated by the electric current is conducted from minor creases to areas with severe creases, thereby eliminating the creases and re-establishing the electrical connection between the electrodes in the HIS (Figure 11c). In practical applications, this unique mechanism enables both touch and display functions on the panel. As shown in Figure 11d,e, HIS has been successfully applied to a portable, reusable, flexible touch panel for wearable human–machine interfaces.

**Figure 12:**
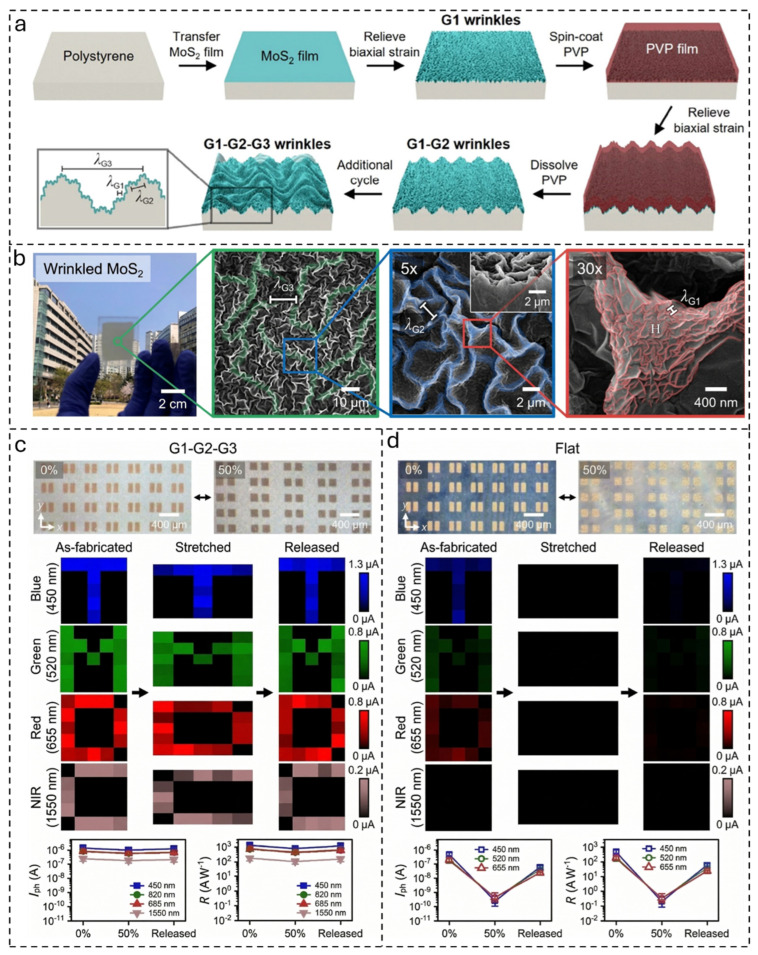
Applications of buckled structures in 2D semiconductors. (**a**) The process of generating layered buckles in solution-processed MoS_2_ nanosheet films. (**b**) Optical images of samples after the formation of third-generation layered buckles. (**c**) Spatial mapping of photocurrent under letter-shaped light illumination at different wavelengths and a summary of the photocurrent and photoresponsiveness of MoS_2_ buckles of different generations under various strain states. (**d**) Spatial mapping of photocurrent under letter-shaped light illumination at different wavelengths and a summary of the photocurrent and photoresponsiveness of flat MoS_2_ films under various strain states. (**a**–**d**) Reproduced with permission [98]. Copyright 2022 American Chemical Society.

Semiconductors are a critical component of electronic devices, but their inherent rigidity limits their application in the field of flexible electronics. Buckled structures can mitigate the stress concentration caused by mechanical deformation and have the potential to provide protection for functional semiconductor layers. As shown in Figure 12a, Rhee et al. [98] fabricated a MoS_2_ conductive layer with a multi-level buckled structure on a PS substrate. The prepared film was able to suppress crack formation even under nearly 100% strain, providing protection for the semiconductor. (Figure 12b). Furthermore, as shown in Figure 12c,d, the introduction of a buckled structure also endows the film with optical responsivity in the visible to near-infrared spectrum, offering a wide detection range (450–1150 nm) and fast response times when applied to stretchable photodetectors. Similarly, Kim et al. [99] fabricated InGaZnO (IGZO) oxide thin-film transistors on an ultra-thin PI film, laminating them onto a pre-stretched elastomer to form a buckled structure. The resulting transistors are capable of suppressing microcracks and electrical degradation caused by long-term cycles of tension and compression.

**Figure 13:**
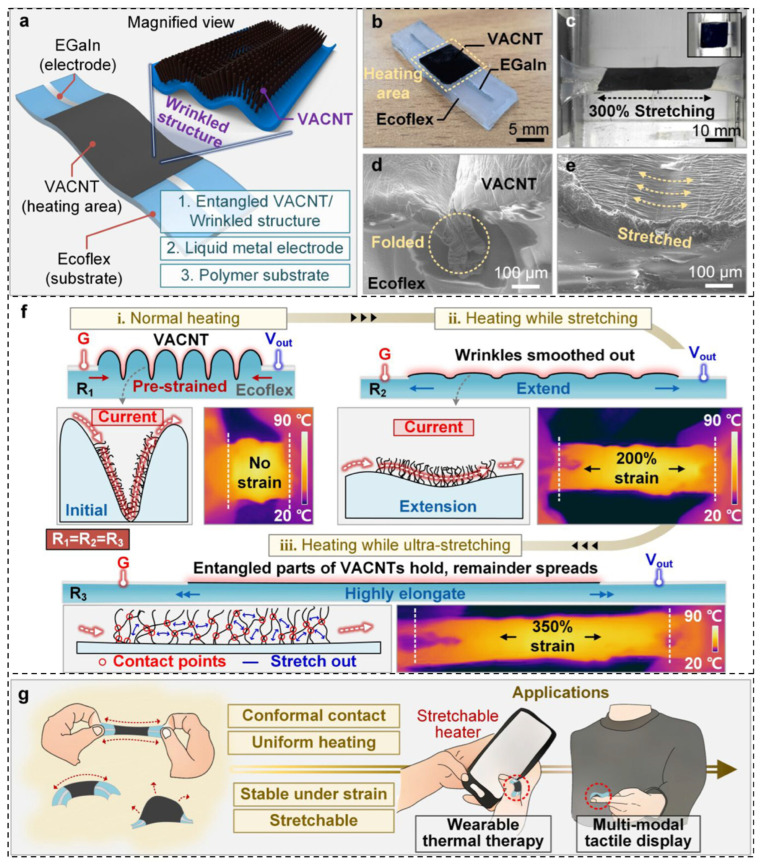
Applications of buckled structures in thermoelectricity. (**a**) Schematic of a stretchable heater based on a buckled structure incorporating VACNTs, comprising a heating element with VACNTs, liquid metal electrodes, and an elastomeric substrate. (**b**,**c**) Optical images of the fabricated heater in its initial state and under 300% stretch. (**d**,**e**) Scanning electron microscope images of the VACNT-integrated buckled structure before and after stretching. (**f**) Drive mechanism of the proposed heater. (**g**) Applications of the stretchable heater in wearable thermal therapy and haptic displays. Reproduced with permission [30]. Copyright 2025 American Chemical Society.

In the field of flexible electric heating devices, buckled structures are used to solve the problem of unstable output in flexible heaters when subjected to tension. The buckled structures can suppress resistance variation and hotspot formation by accommodating tensile strain through the unfolding of wavy conductive paths. Furthermore, unfolding of the buckled structures enlarges the specific surface area, thereby facilitating light absorption and thermal transfer efficiency in electrothermal devices. As shown in Figure 13a, Bae et al. [30] integrated vertically aligned carbon nanotubes (VACNTs) bundles onto the surface of a pre-stretched Ecoflex elastomer to create a buckled structure. The fabricated electric heating elements exhibit high tensile stability and excellent service life. Within the 0–350% strain range and after 10,000 cycles at 200% strain, the temperature fluctuations remained below 5%. This stable heat output makes it suitable for thermal therapy of finger joints (Figure 13b–e). As shown in Figure 13f, the flexible surface of the heater forms a buckled morphology with the transferred VACNT sheet. When strain is applied to the heater, the buckled structure stretches, but the VACNTs remain unchanged, thereby ensuring uniform heating performance. When the device is stretched to its ultimate strain value, the VACNT sheet expands along the direction of the applied strain within each optical fiber of the network during the stretching process. Furthermore, as illustrated in Figure 13g, the produced heater can be combined with a pneumatic pressure unit for use in various applications, ranging from thermal therapy devices to haptic displays that can simulate pressure and heat simultaneously.

In wireless communication applications, buckled structures have attracted more attention due to their excellent stretchability and ability to conform to complex surfaces. The buckled structures stabilize the resonant frequency during stretching by keeping the effective electrical length nearly constant. Wang et al. [100] coated the surface of a pre-inflated catheter with a carbon nanotube film and induced a buckled structure by deflating it. This structure is capable of achieving a volumetric strain of up to 7470% during inflation and deflation and shows stable electrical conductance and surface wettability during large-strain inflation/deflation cycles. The researchers successfully used this technology to achieve remote control of the inflation, deflation, and movement of an inflatable radiofrequency antenna.

## 4. Conclusions and Perspectives

The design of buckled structures using geometric mechanisms, as a classic strategy for imparting flexibility and stretchability to rigid materials, has become a major area of research in the field of flexible electronics. Leveraging the strain absorption characteristics of buckled structures enables electronic devices to achieve high flexibility while simultaneously acquiring additional functionalities, including hydrophobicity and high optical transparency. These combined advantages drive the continuous expansion of application scenarios for FEDB, gradually encompassing emerging fields such as health monitoring, human–machine interaction, soft medical devices, and underwater sensing. However, despite the progress achieved by FEDB, practical applications of these devices still face a series of critical challenges:

(1) How can the stability and structural durability of FEDB be ensured during long-term operation whilst maintaining their excellent electrical performance?

With the development of FEDB, the application scenes of FEDB are expanding. The researchers must consider the stability of flexible electronic devices into account when they are being used in practice. Although the incorporation of a buckled structure can impart excellent flexibility and even stretchability to electronic devices, stress concentration during long-term use inevitably leads to delamination between the substrate and the conductive material, which results in device failure. While existing research has largely focused on strategies such as creating multi-level buckles and interface locking to enhance the service life of buckled structures, chemical modification and interface reinforcement often compromise the device’s electrical performance. For example, poor interfacial adhesion between the conductive AgNWs/PEDOT: PSS layer and the PDMS substrate can result in gradual resistance drift after hundreds of stretch-release cycles [28,29]. Even with hierarchical buckles, stress concentration at the crests and valleys can cause local delamination, as observed in sheath-core fibers under 1000% strain [32]. Therefore, achieving long-term stable device operation requires selecting suitable materials and constructing highly stable buckled structures while maintaining electrical characteristics.

(2) Although the existing buckled system can effectively improve the device’s stretchability and electrical conductivity, its ability to withstand extreme environments still requires systematic investigation.

In practical application environments, external conditions such as temperature, humidity, and surrounding media undergo continuous dynamic changes. Substrate materials are susceptible to environmental influences and subsequent structural degradation. Meanwhile, the gradual expansion of FEDB into specialized application scenarios, including the human body, underwater environments, and the deep ocean and outer space, demands the long-term stable operation of electronic devices under stringent conditions such as high pressure, corrosive atmospheres, and extreme temperatures and humidity levels. An example is the underwater strain sensor [27], where the Ecoflex/PDMS substrate must resist water penetration and salt corrosion while maintaining buckled morphology. However, prolonged immersion causes substrate swelling and a shift in the critical buckling strain, degrading the sensor’s negative-resistance response. Therefore, optimizing the performance of flexible electronic devices to accommodate these increasingly severe environmental requirements is a primary challenge.

(3) How to precisely control the microscopic morphology of the buckles, including their wavelength, amplitude, and orientation, to optimize interfacial adhesion properties and enable the device to adapt efficiently to human tissue and complex 3D surfaces?

Monitoring human physiological signals is a crucial application for FEDB, requiring FEDB to conform closely to the dynamically deforming, curved surfaces of human skin. Although existing buckled structures generally meet the requirements for conventional skin-adhering sensors, there is still space for improvement in terms of interface conformability when dealing with complex surfaces subject to large deformations, such as joints and buckled skin. For conformal attachment to curved skin, the CBH sensor employs a multi-level buckle design inspired by serpentine and butterfly wings, yet when applied to joints with large out-of-plane deformation, the buckled orientation fails to follow the local curvature gradient, causing partial detachment and signal hysteresis [69].

(4) Currently, the production of FEDB is largely limited to laboratory-scale operations. The key challenge hindering their practical application is the lack of laboratory-scale, low-cost, scalable mass production technologies.

Streamlined, rapid, low-cost, and large-scale fabrication of FEDB is critical for commercial deployment. For instance, the prestretch-release method used in most studies requires manual pre-straining and careful strain release, which are difficult to control uniformly over large areas [28,60].

In summary, the classic geometric strategy of buckled structures has effectively promoted developments in the performance and functional expansion of flexible electronic devices. In turn, the diverse application of flexible electronics has driven the refinement and functional optimization of buckled structural designs. With flexible electronic devices continuing to gain traction in emerging fields such as health monitoring, human–computer interaction, and underwater sensing, their future development potential and market value are vast. By integrating theoretical modeling and finite element analysis, the structural wavelength, amplitude, orientation, and curvature of FEDB could be precisely controlled, which enables FEDB to meet the requirements of different devices for stretchability, sensitivity, interfacial conformity, and functional stability. Meanwhile, the development of low-cost, large-area, and continuous fabrication technologies will help improve the uniformity and batch-to-batch reproducibility of buckled structures. Furthermore, further integration with sensing, power supply, communication, and signal processing modules is expected to promote the practical application of FEDB in areas such as electronic skin, wearable healthcare, implantable devices, soft robotics, and adaptive optoelectronic systems.

## Figures and Tables

**Table 1:** Young’s modulus, fracture strain, and electrical conductivity of common conductive materials.

Material	Young’s Modulus [GPa]	Fracture Strain (%)	Electrical Conductivity (S/m)	Ref
AgNWs	80–90	4	6.3 × 10^9^	[42,43]
Au	70–80	>5	4 × 10^9^	[44,45]
Monolayer graphene	≈100	25	≈10^8^	[46,47]
CNTs	147–270	<15	SWCNTs ≈ 10^6^–10^8^ MWCNTs ≈ 10^5^–10^7^	[48,49,50]
PEDOT: PSS	1–2.7	3–5	4.38 × 10^5^	[9]
PPy	≈3	≈9	8 × 10^4^	[51]
Cu	70–100	7.2	5.96 × 10^9^	[52,53]
MXene	≈330	5–6	1.1 × 10^6^	[54,55]
MoS_2_	170–370	6–11	2H phase: 10^−2^–10^−1^	[56,57]
			1T phase: 10^5^–10^6^	

**Table 2:** Different application scenarios with their corresponding performance requirements.

Application Scenarios	Facing Challenges	Corresponding Performance Requirements
Wearable Applications	Interface Delamination and Performance Degradation	Softness, High Stretchability, Long-term Cyclic Stability, Breathability
Underwater Applications	Seawater Corrosion and Signal Attenuation in Underwater Environments	Hydrophobicity, Waterproofness, Environmental Stability (Salt/Water Resistance, Anti-Corrosion)
In Vivo Applications	Immune Rejection and Biofouling Caused by Long-term Implantation	Biocompatibility, Reliable Encapsulation, Biodegradability, Minimal Immune Response
Human–Machine Interfaces	Difficulty in Maintaining Conformal Attachment on Complex 3D Human Surfaces	High Sensitivity, Fast Response Time, Conformal Contact with Skin
Soft Robotics	Material Fatigue and Fracture Failure Caused by Continuous Large-Amplitude Actuation	High Deformability, Durability Under Repeated Actuation, Rapid Response
Energy Device	Formation of Microcracks in the Active Layer and Destruction of Conductive Networks During Mechanical Deformation	Large Surface Area, High Cyclic Stability, Efficient Energy Conversion

**Table 3:** Comparison of electrical properties and stretchability for stretchable electrodes.

Material	Buckled Formation Principles	Initial Electrical Properties	Mechanical Durability	Quality Factor (Q)	Year	Ref
Carbon nanotube flakes/SEBS rubber fibers	Prestretch-release method	26.1 Ω/cm	Under a strain of 3000%, the change in resistance is less than 5.01%	598	2015	[32]
PEDOT/PSS/PBP/TPE	Prestretch-release method	88~95 S/m	Under a strain of 680%, the change in resistance is less than 4%	178.25	2020	[67]
PPy/WPU/PU multi-filament core	Prestretch-release method	100 S/m	Under a strain of 600%, the change in resistance is less than 0.66	9.09	2022	[80]
Ag nanowires/PEDOT: PSS/ionic liquid/PDMS/TPU	Prestretch-release method and solvent annealing	33.5 Ω/sq	Under 80% strain, the rate of change in resistance is 8%	10	2023	[59]
Ag film/PEDOT: PSS/PDMS	Prestretch-release method	0.91 Ω/sq	Under 80% strain, the change in resistance is close to 0%	/	2025	[81]
Platinum nanomembrane/PDMS	Prestretch-release method	4.1 × 10^5^ Ω	Under a strain of 40%, the rate of change in resistance is less than 3%	/	2025	[82]
PPy/PEDOT/SEBS/PDMS	Prestretch-release method	358 S/m	Under a strain of 200%, the change in resistance is less than 0.36	1111	2025	[29]
PHEA-co-PHEAA flexible hydrogel/PPy	Prestretch-release method	8.15 × 10^3^ S/m	Under 100% tensile strain, the change in electrical resistance is 9.2%	/	2025	[83]
AgNWs/PEDOT: PSS/PDMS	Mold	5.1~5.3 Ω/sq	Under a strain of 10%, the change in resistance is less than 0.6%	16.67	2025	[28]

**Table 4:** Comparison of the sensing characteristics and mechanical durability of flexible strain sensors.

Material	Buckled Formation Principles	Initial Electrical Properties	Mechanical Durability	Gauge Factor (GF)	Year	Ref
Double-walled carbon nanotube/rubber composite fibers	Prestretch-release	1.8 × 10^4^ Ω	1000 cycles at 600% strain	0.14	2017	[84]
Au/PDMS	Mold/Prestretch-release	/	10,000 cycles at 5% strain	2557.71	2022	[87]
Reduced graphene oxide/natural latex rubber substrate	Prestretch-release	2.668 × 10^5^–2.6696 × 10^8^ Ω/sq	600 cycles at 15% strain	49.5	2022	[88]
MXene film/PDMS	Prestretch-release	/	1000 cycles at 50% strain	45–117	2023	[68]
Acrylic/Au/Ecoflex	Prestretch-release	Low	10,000 cycles at 1% strain	3627	2023	[86]
Ag/PDMS	Prestretch-release	/	6000 cycles at 2.5% strain	45.6–4125	2023	[89]
MXene/graphite/PDMS	Prestretch-release	/	over 3500 cycles at 120% strain	0.24–1.47	2024	[60]
CNT/MXene/PDMS	Prestretch-release	1.5 × 10^3^ Ω	1000 cycles at 30%, 50%, and 80% strain	283.4–1756.9	2024	[69]
AgNWs/PDMS	Mold	32~168 Ω/sq	500 cyclic tensile tests at 20% strain	14.52	2024	[90]
Cr/Ag/Cr/PDMS	Prestretch-release	/	22,000 cycles at 4% strain	9.2 × 10^6^	2025	[91]
CNTs/PDMS/candle soot nanoparticles	Prestretch-release	/	5000 cycles at 60% strain	1.06	2025	[27]

## Data Availability

The data that support the findings of this study are available from the corresponding author upon reasonable request.
